# Supplementary material for: Worsening Glycemia Increases the Odds of Intermittent but Not Persistent Staphylococcus aureus Nasal Carriage in Two Cohorts of Mexican American Adults
Source: Microbiol Spectr. 2022 May 18;10(3):e00009-22. doi: 10.1128/spectrum.00009-22 (PMC9241628; doi:10.1128/spectrum.00009-22)
Supplement: SUPPLEMENTAL FILE 1 — Supplemental material. Download spectrum.00009-22-s001.pdf, PDF file, 1.1 MB [file spectrum.00009-22-s001.pdf]

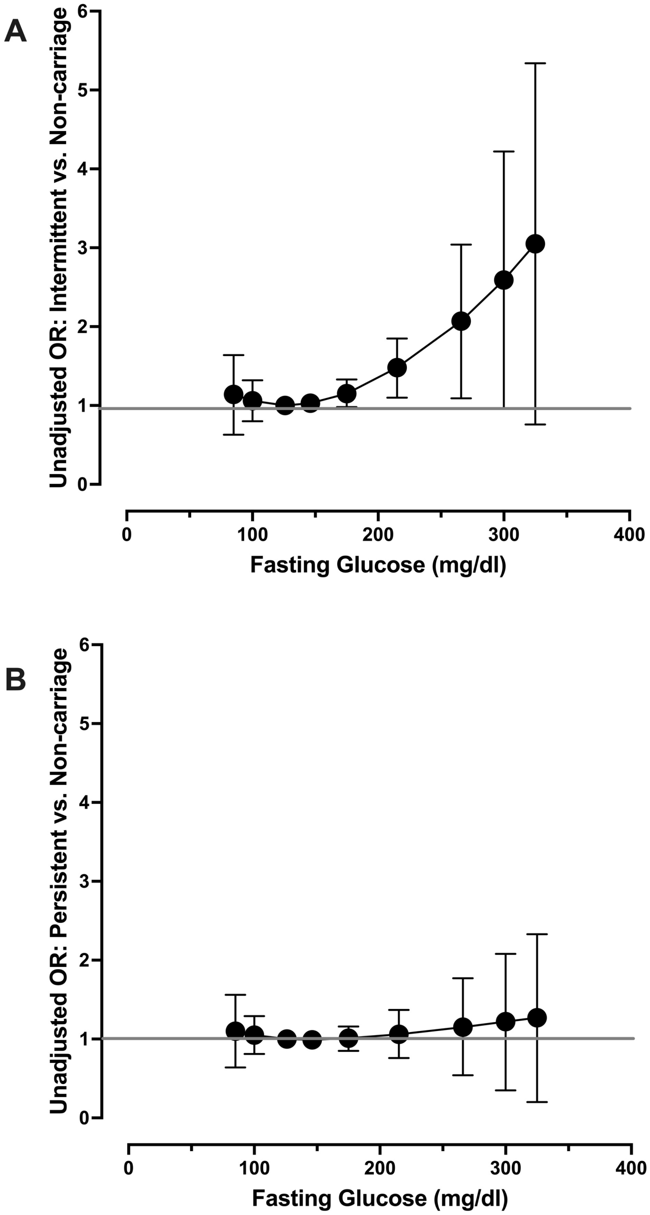

Figure S1. Unadjusted odds of intermittent (A) and persistent carriage (B), compared to non-carriers, respectively, across the spectrum of fasting glucose (mg/dL) in the primary cohort. The unadjusted odds of intermittent carriage compared to non-carriage across the spectrum of fasting plasma glucose modeled with three-knot restricted cubic splines (knots at 125, 171, 225 mg/dL) and with a fasting glucose value of 126 mg/dL (the clinical cut-point for diabetes) as the

referent value **(A)**. For comparison purposes, **(B)** models the same relationship for persistent carriers compared to non-carriers.

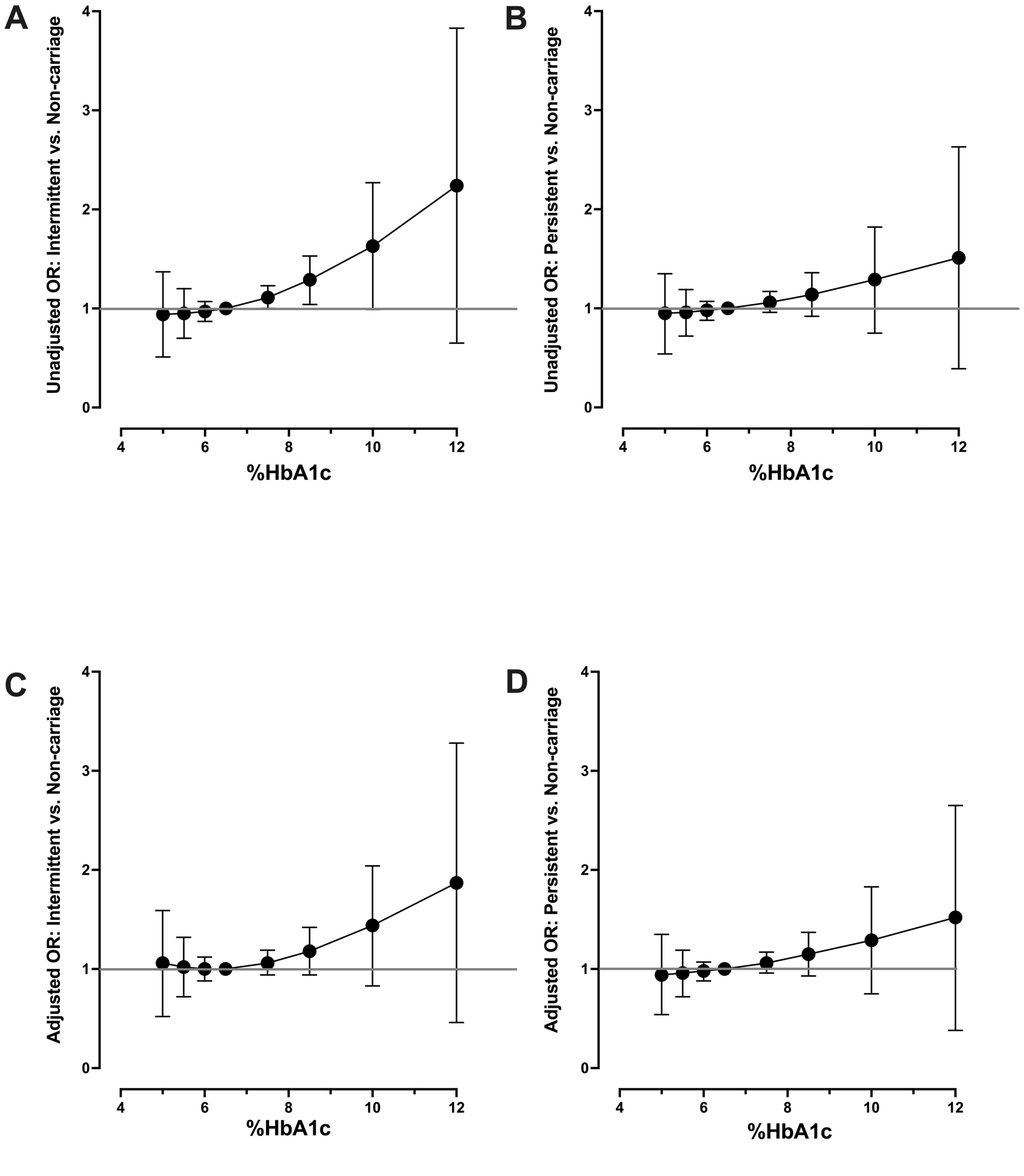

Figure S2. Unadjusted (A, B) and adjusted odds (B, C) of intermittent (A, C) and persistent carriage (B, D), compared to non-carriers, respectively, across the spectrum of percent glycated

hemoglobin A1c (%HbA1c) in the primary cohort. The odds of intermittent carriage compared to non-carriage across the spectrum of %HbA1c modeled with three-knot restricted cubic splines (knots at 5.1%, 5.8%, 9.2%) with 6.5% (the clinical cut-point for diabetes) as the referent value. **(A)** and **(B)** show unadjusted odds of intermittent and persistent carriage, respectively, across the spectrum of %HbA1c. **(C)** shows the adjusted odds of intermittent carriage across %HbA1c, adjusted for use of DPP4 inhibitors, BMI, total cholesterol, and use of an antibiotic in the past 30 days. **(D)** depicts the adjusted odds of persistent carriage across %HbA1c adjusted for number of rounds of antibiotics used in the 12 months. %HbA1c was not selected for inclusion into the multivariable models and was not significantly associated with persistent carriage.

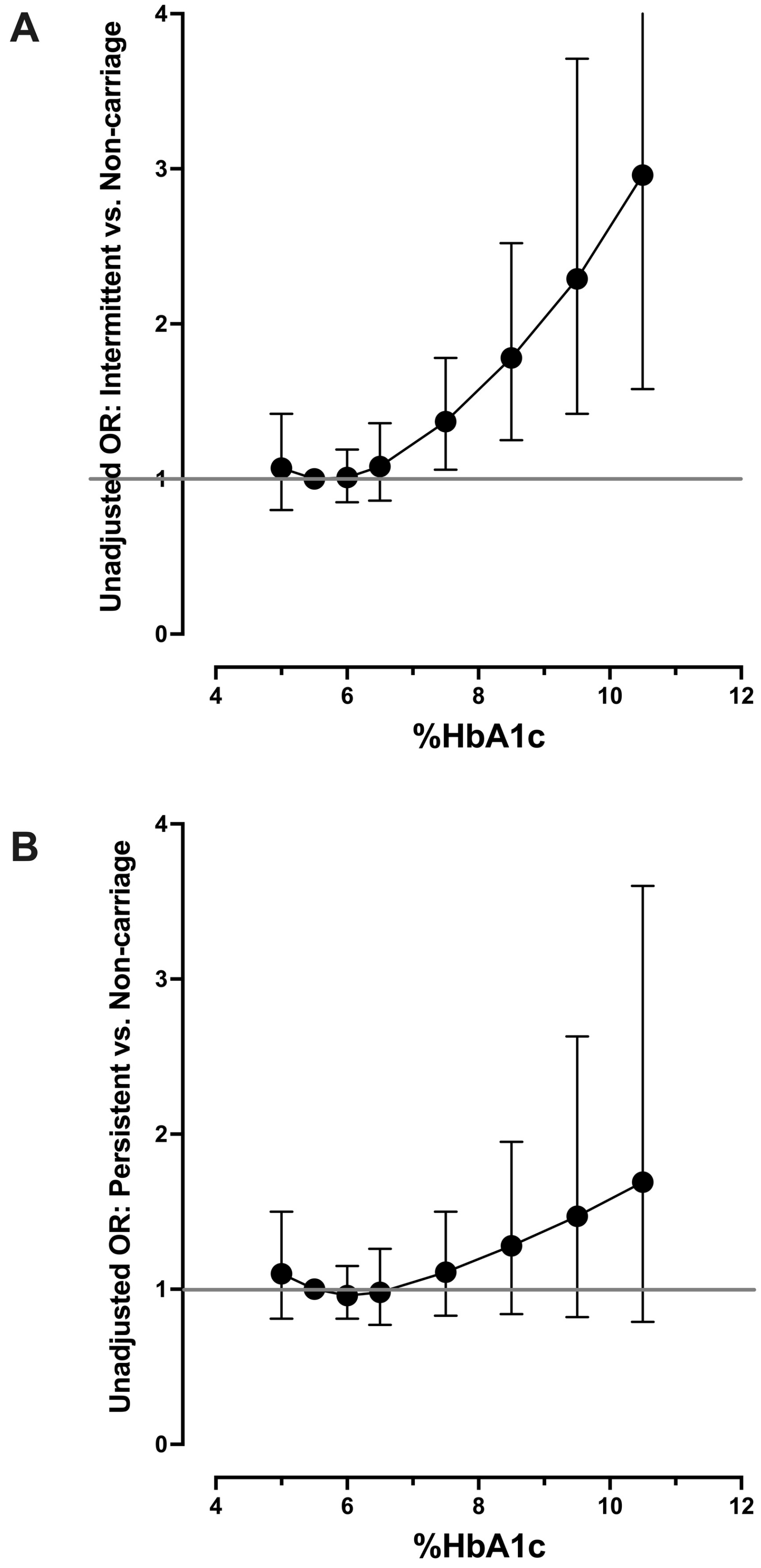

A
Unadjusted OR: Intermittent vs. Non-carriage
0
1
2
3
4
4
6
8
10
12
%HbA1c
B
Unadjusted OR: Persistent vs. Non-carriage
0
1
2
3
4
4
6
8
10
12
%HbA1c

Figure S3. Unadjusted odds of intermittent (A) and persistent carriage (B) compared to non-carriers, respectively, across the spectrum of % glycated hemoglobin A1c (%HbA1c) in the replication cohort. The unadjusted odds of intermittent carriage compared to non-carriage across the spectrum of %HbA1c modeled with three-knot restricted cubic splines (knots at 5%, 5.5%, 7.3%) with 5.5% as the referent value **(A)**. For comparison purposes, **(B)** models the same relationship for persistent carriers compared to non-carriers.
